# Supplementary material for: Differential Behavior of Conformational Dynamics in Active and Inactive States of Cannabinoid Receptor 1
Source: J Phys Chem B. 2024 Aug 22;128(35):8437–47. doi: 10.1021/acs.jpcb.4c02828 (PMC11382280; doi:10.1021/acs.jpcb.4c02828)
Supplement: Supplementary file 4 — jp4c02828_si_004.pdf [file jp4c02828_si_004.pdf]

Supporting Information: Differential Behavior of Conformational Dynamics in Active and Inactive States of Cannabinoid Receptor 1

Ugochi H. Isu, Adithya Polasa, and Mahmoud Moradi*

Department of Chemistry and Biochemistry, University of Arkansas, Fayetteville, AR 72701

E-mail: moradi@uark.edu

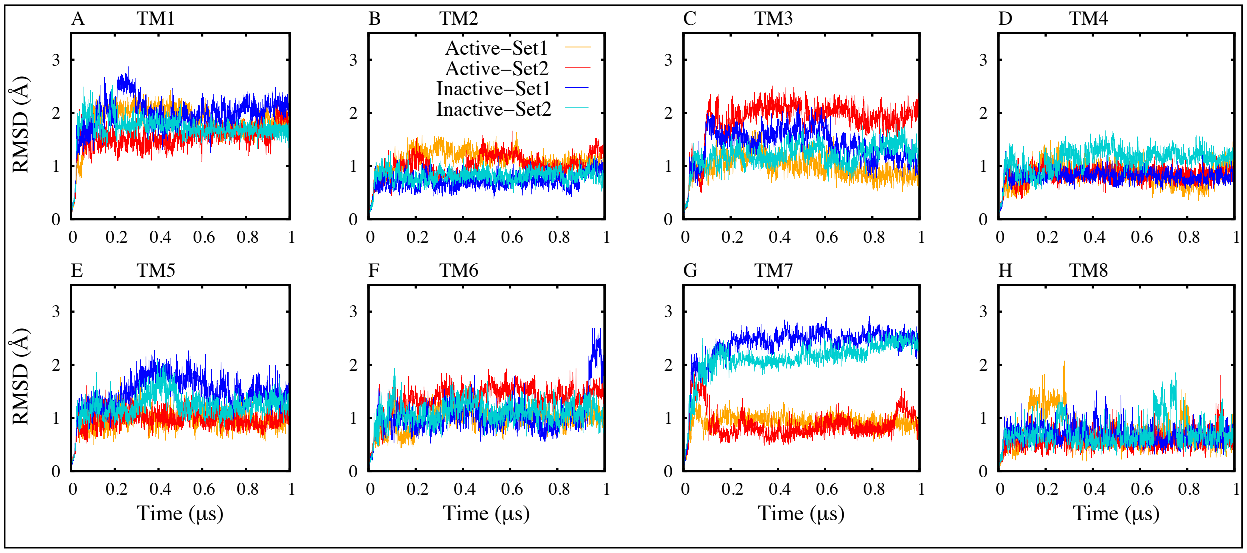

Fig. S1. **C_α atom RMSD analysis of TM helices with respect to the initial model across Simulation Sets 1 and 2.**

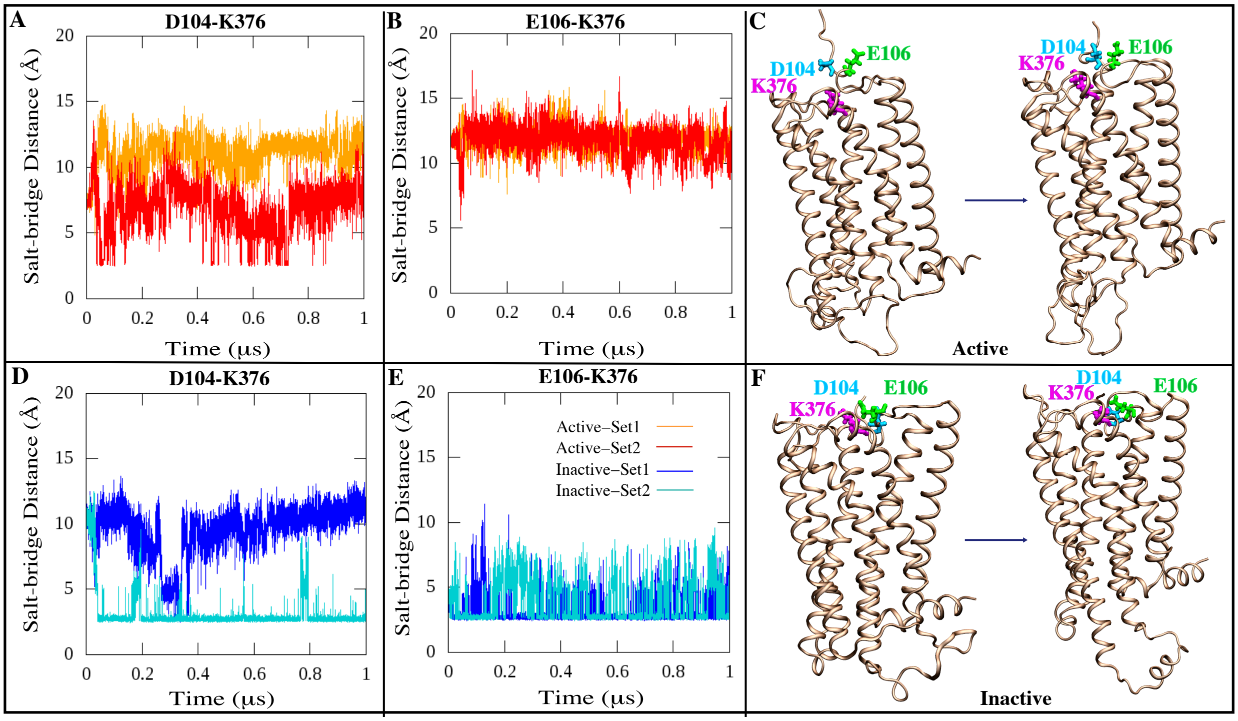

Fig. S2. Network of salt bridge interactions partially present in inactive state but absent in active state. Salt bridges between K376 (magenta) and D104 (cyan)/E106 (green). (A, D) Time series plots of the K376-D104, (B, E) Time series plots of the K376-E106, donor-acceptor salt bridge distances of the active and inactive states for both simulated replicates. (C, F) Graphical illustration of salt-bridge interactions between the K376-D104/E106 in the active and inactive states.

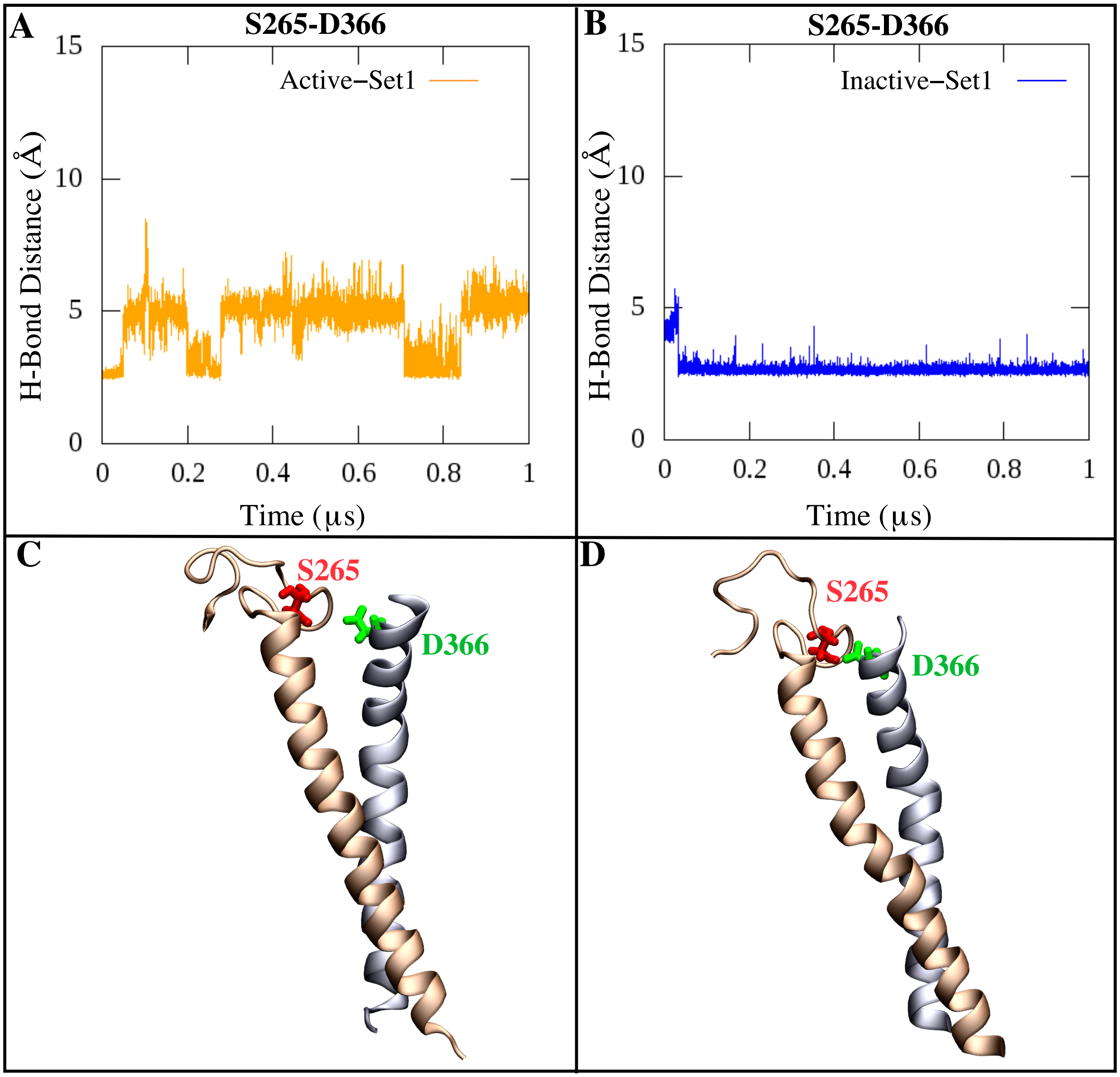

Fig. S3. Differential hydrogen bonding between S265 and D366 in (A) active and (B) inactive models of set 1 CB1 simulation.

Supporting Movies

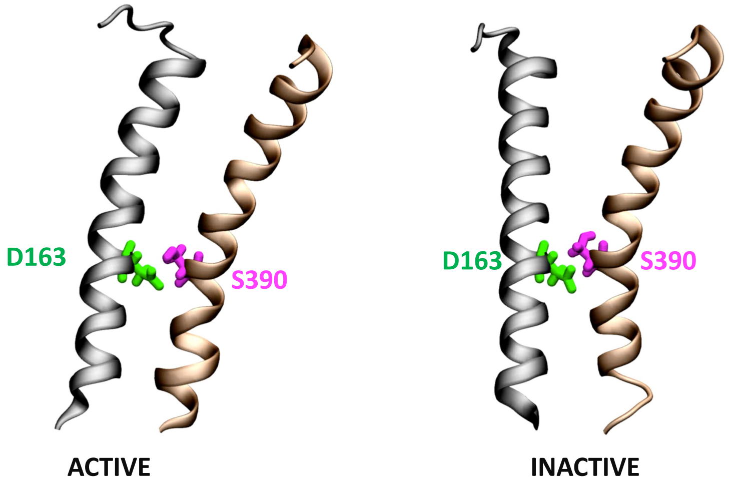

Mov. S1. Movie visualizing H-bond interaction between D163 on TM2 and S390 on TM7.

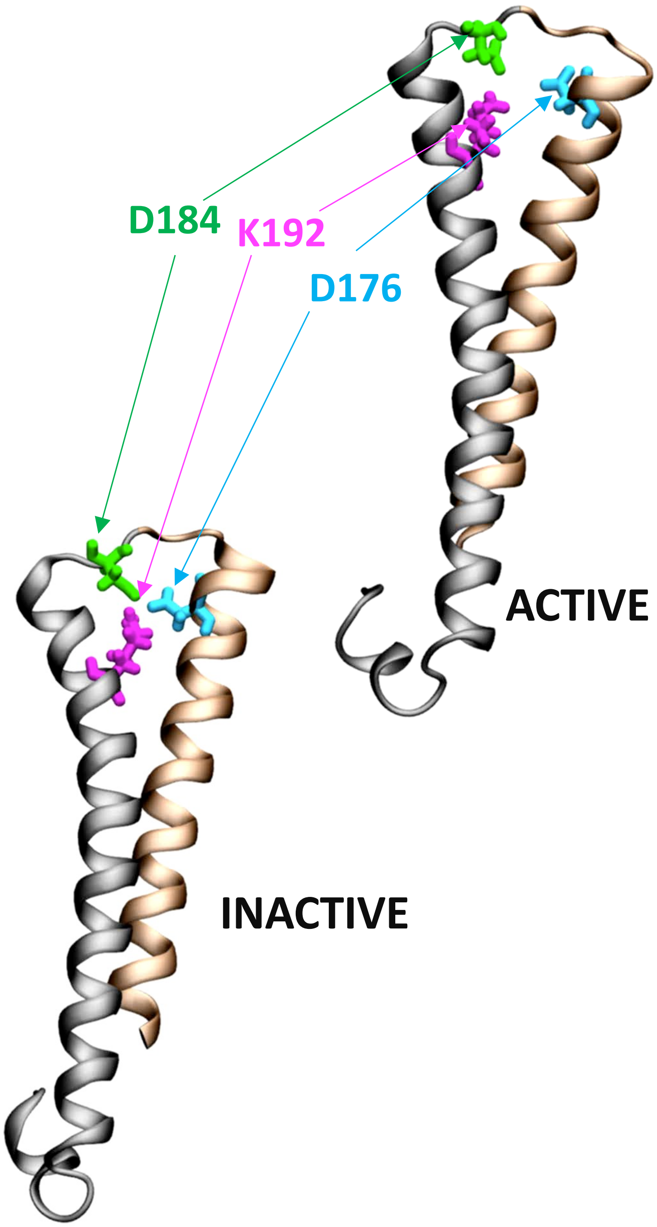

Mov. S2. Salt–bridge interaction network between K192 and D176/D184. Positively charged K192 (magenta) fluctuates between negatively charged D176 and D184 (cyan and green) respectively.

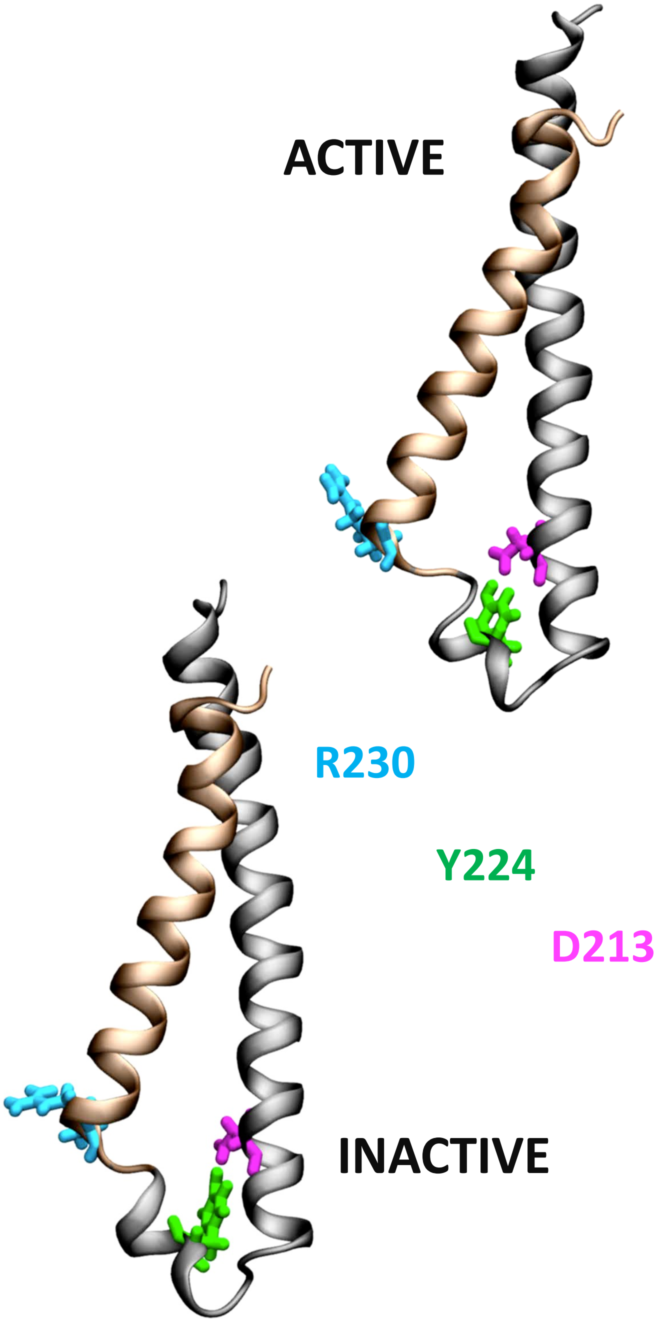

Mov. S3. Movie showing interactions between D213, Y224, and R230.

TM	TM	active			inactive		
		initial value	average	s.d.	initial	average	s.d,
1	2	6.7	7.1	0.4	7.9	7.3	0.8
1	3	19.1	20.0	0.5	21.5	20.2	0.8
1	4	22.2	23.3	0.4	23.8	23.4	0.8
1	5	24.8	26.1	0.7	26.3	25.7	0.7
1	6	19.8	20.3	0.5	19.4	19.6	0.6
1	7	10.2	10.1	0.3	10.7	10.4	0.6
2	3	16.7	17.3	0.4	18.3	18.1	0.4
2	4	17.4	18.1	0.5	17.8	18.5	0.3
2	5	23.7	24.3	0.6	24.8	25.1	0.6
2	6	20.2	20.2	0.5	19.5	20.4	1.0
2	7	11.7	11.6	0.3	13.4	13.3	0.8
3	4	9.1	9.9	0.4	9.1	9.6	0.3
3	5	11.7	11.6	0.4	11.3	11.5	0.4
3	6	15.6	15.3	0.6	13.6	14.9	0.5
3	7	17.0	17.1	0.4	18.6	18.5	0.6
4	5	18.9	19.4	0.4	18.0	19.0	0.5
4	6	22.8	23.4	0.4	20.0	22.3	0.8
4	7	22.3	23.0	0.3	23.1	24.1	0.4
5	6	9.2	9.8	0.3	8.7	9.5	0.3
5	7	17.7	18.6	0.6	18.7	19.1	0.5
6	7	10.6	10.9	0.4	10.3	10.7	0.3

Table S1. Interhelical distances (in $\mathring{A}$) based on the mass center of C_α atoms of each TM helix determined from the initial model (initial value) and the average over the entire trajectories of both sets of each system (active vs inactive) along with the estimated standard deviation (s.d.).
